# Stem/Progenitor Cell Niches Involved in Hepatic and Biliary Regeneration

**DOI:** 10.1155/2016/3658013

**Published:** 2016-01-10

**Authors:** Guido Carpino, Anastasia Renzi, Antonio Franchitto, Vincenzo Cardinale, Paolo Onori, Lola Reid, Domenico Alvaro, Eugenio Gaudio

**Affiliations:** ^1^Department of Movement, Human and Health Sciences, Division of Health Sciences, University of Rome “Foro Italico”, Piazza Lauro De Bosis 6, 00135 Rome, Italy; ^2^Department of Anatomical, Histological, Forensic Medicine and Orthopedics Sciences, Sapienza University of Rome, Via Borelli 50, 00161 Rome, Italy; ^3^Eleonora Lorillard Spencer-Cenci Foundation, Piazzale Aldo Moro 5, 00185 Rome, Italy; ^4^Department of Medico-Surgical Sciences and Biotechnologies, Sapienza University of Rome, Corso della Repubblica 79, 04100 Latina, LT, Italy; ^5^Department of Cell Biology and Physiology, Chapel Hill, NC 27599, USA; ^6^Program in Molecular Biology and Biotechnology, Chapel Hill, NC 27599, USA; ^7^Lineberger Comprehensive Cancer Center, Chapel Hill, NC 27599, USA

## Abstract

Niches containing stem/progenitor cells are present in different anatomical locations along the human biliary tree and within liver acini. The most primitive stem/progenitors, biliary tree stem/progenitor cells (BTSCs), reside within peribiliary glands located throughout large extrahepatic and intrahepatic bile ducts. BTSCs are multipotent and can differentiate towards hepatic and pancreatic cell fates. These niches' matrix chemistry and other characteristics are undefined. Canals of Hering (bile ductules) are found periportally and contain hepatic stem/progenitor cells (HpSCs), participating in the renewal of small intrahepatic bile ducts and being precursors to hepatocytes and cholangiocytes. The niches also contain precursors to hepatic stellate cells and endothelia, macrophages, and have a matrix chemistry rich in hyaluronans, minimally sulfated proteoglycans, fetal collagens, and laminin. The microenvironment furnishes key signals driving HpSC activation and differentiation. Newly discovered third niches are pericentral within hepatic acini, contain Axin2+ unipotent hepatocytic progenitors linked on their lateral borders to endothelia forming the central vein, and contribute to normal turnover of mature hepatocytes. Their relationship to the other stem/progenitors is undefined. Stem/progenitor niches have important implications in regenerative medicine for the liver and biliary tree and in pathogenic processes leading to diseases of these tissues.

## 1. Introduction

The biliary tree is a complex network of interconnected ducts, which drain bile into the duodenum [1]. It can be divided into intrahepatic and extrahepatic portions. The intrahepatic biliary tree is composed of small (canals of Hering, bile ductules, interlobular ducts, and septal ducts) and large (area and segmental) bile ducts (BDs) [2, 3].

**Figure 1:**
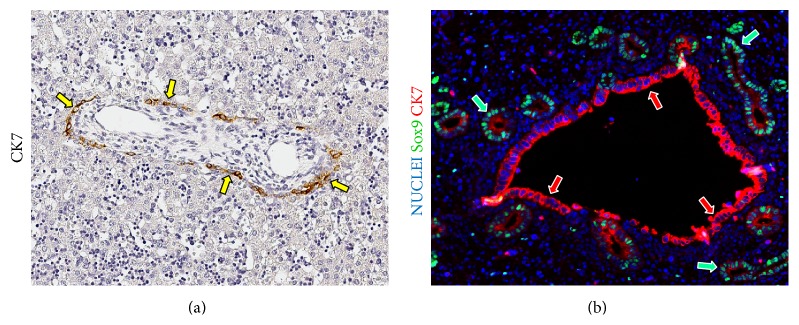
Embryology of stem/progenitor cell niches within the biliary tree. (a) Human fetal livers (20th week gestational age). Immunohistochemistry for cytokeratin (CK) 7. The ductal plate is present around portal tracts and contains CK7+ cells (arrows). Original Magnification: 10x. (b) Human fetal hepatic duct at the liver hilum (20th week gestational age). Immunofluorescence for Sox9 and CK7. Peribiliary glands (green arrows) derive from outpouches of the surface epithelium (red arrows) of the hepatic duct. Original Magnification: 20x.

Cholangiocytes are specialized and heterogeneous epithelial cells, lining BDs [4]. In particular, small cholangiocytes line small intrahepatic BDs, while large cholangiocytes line large intrahepatic and extrahepatic BDs [4]. Interestingly, small and large cholangiocytes differ on the basis of their dimensions, ultrastructure (absence or presence of primary cilia), functions, and proliferative capabilities [4–7]. In addition, small and large ducts have a separate embryological origin. Ductal plates, found in fetal and neonatal livers, give rise to small intrahepatic BDs, whereas the elongation and molding of the hepatic diverticulum give rise to the large intrahepatic and extrahepatic BDs (Figure 1) [2, 8].

In adults, there are multiple niches of stem/progenitor cells residing in different locations along the human biliary tree and niches found within the liver parenchyma. Those within the biliary tree are found in peribiliary glands (PBGs) and contain especially primitive stem cell populations, expressing endodermal transcription factors relevant to both liver and pancreas, pluripotency genes, and even markers indicating a genetic signature overlapping with that of intestinal stem cells [9]. The biliary tree stem/progenitors (BTSCs) support the renewal of large intrahepatic and extrahepatic BDs [1]. Canals of Hering (bile ductules), the smaller branches of the biliary tree, are niches containing hepatic stem/progenitors (HpSCs) and participating in the renewal of the small intrahepatic BDs and in the regeneration of liver parenchyma [10, 11]. A third set, found pericentrally within the liver acinus, is newly discovered and is comprised of Axin2+ unipotent hepatocytic progenitors that are linked on their lateral borders to the endothelia forming the central vein and constitute precursors to the mature hepatocytes in normal liver turnover and mild regenerative responses [12].

## 2. Biliary Tree Stem/Progenitor Cells (BTSCs)

**Figure 2:**
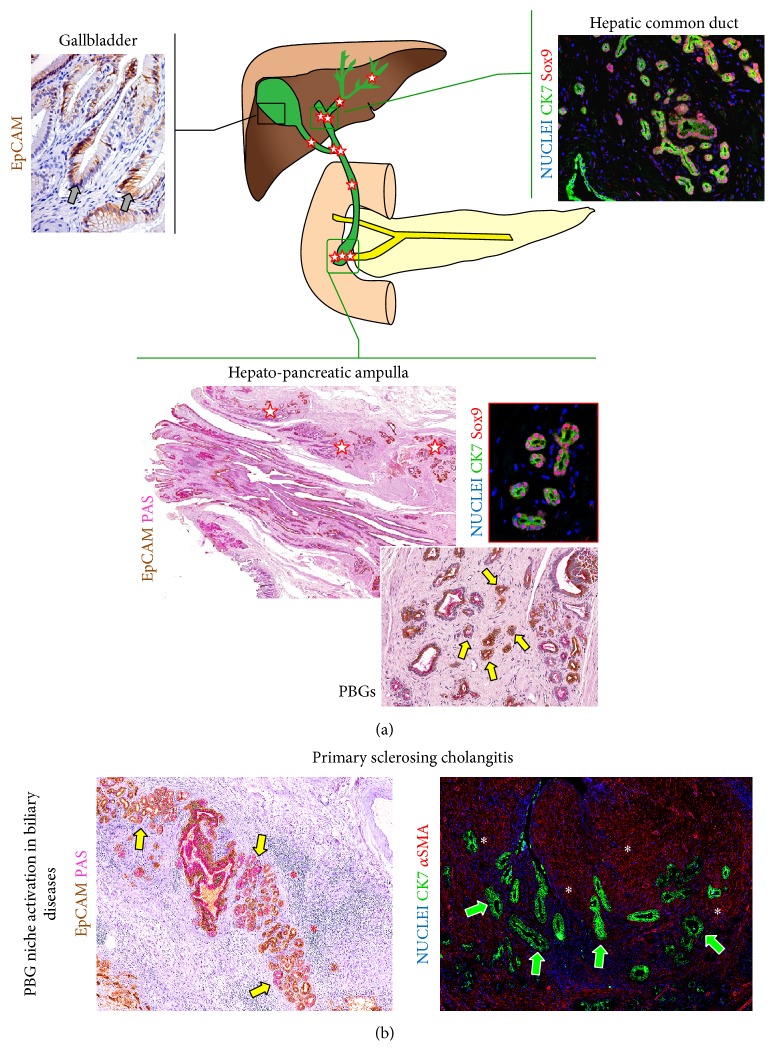
Peribiliary glands (PBGs) are the niche of Biliary Tree Stem Cells (BTSCs). (a) PBGs are glands located within the lamina propria of the extrahepatic and large intrahepatic bile ducts (yellow arrows). PBG distribution varies along the biliary tree, and PBGs are mostly found in the hepatopancreatic ampulla (white stars) and in branching sites of the biliary tree. PBGs are not present in gallbladder, but a BTSC-like compartment is located in the epithelial crypts (gray arrows). PBGs are composed of Sox9+ BTSCs. (b) Primary sclerosing cholangitis is characterized by the inflammation of duct walls (red asterisks) and PBG hyperplasia (yellow arrows). PBGs are involved in biliary fibrosis and are surrounded by *α*-smooth muscle actin (*α*-SMA) fibrogenetic cells (white asterisks). Immunohistochemistry for Epithelial Cell Adhesion Molecule (EpCAM) was counterstained with Periodic acid-Schiff (PAS). Immunofluorescence for cytokeratin (CK) 7, Sox9, and *α*-SMA are included.

Beside HpSCs within the smaller branches of the biliary tree, a second stem/progenitor cell niche is located along large intrahepatic and extrahepatic BDs [13]. BTSCs represent a stem/progenitor cell compartment located within PBGs (Figure 2) [14]. PBGs are located in the lamina propria of large intrahepatic and extrahepatic BD walls and are communicating with the duct lumen [2, 15]. BTSCs are composed of heterogeneous populations characterized by phenotypic traits of ventral endoderm, expressing typical transcription factors (SOX9, SOX17, and PDX1), surface (EpCAM, LGR5, and/or CD133), and cytoplasmic markers (CK7, CK19) [1]. As a restricted population, a subset of the BTSCs (nearly 10%) expresses pluripotency markers such as OCT4, SOX2, NANOG, SALL4, and KLF 4/5 and their* in vitro* capabilities qualify them as primitive true stem cells [13]. BTSCs have multipotent capabilities and can differentiate towards functional hepatocytes, mature cholangiocytes, and pancreatic endocrine cells [14]. Whether or not they can give rise to acinar cells is yet to be determined.

The distribution of PBGs is not uniform, varying along the biliary tree: PBGs are mostly found in the hepatopancreatic ampulla and are less numerous in common bile duct [13, 14]. PBGs are not present in gallbladder, but a BTSC-like compartment is located in the epithelial crypts [16]. Interestingly, a proximal-to-distal axis in a maturational lineage is found along the biliary tree [13, 17]. The highest numbers of the very primitive BTSCs, ones that are precursors to both pancreas and liver, are located primarily within PBGs near the hepatopancreatic ampulla; with progression towards the liver and intrahepatic BDs, there is a decline in the numbers of these very primitive BTSCs and gradual increase in stem/progenitors for cells with hepatic fates [13].

Furthermore, the PBG niches are characterized by the presence of a radial axis in maturational lineages [13]: the more undifferentiated and proliferating cells are located at the bottom of the glands, near the fibromuscular layer at the centers of the ducts; the cells with a committed phenotype are found in the middle and the fully differentiated cells are in continuum with the surface epithelium [13].

Recently, several lines of evidence in rodents and in humans indicate that BTSCs and their niches are implicated in the turnover of the surface epithelium of large intrahepatic and extrahepatic BDs [18, 19]. An experimental mouse model confirmed the existence within the PBGs of multiple cell lineages and the proliferation of cells within PBGs after duct injury [18]. Moreover, in human extrahepatic BDs affected by cholangitis, proliferating progenitor cells were mainly located in PBGs, which can be considered a local stem/progenitor cell niche [19].

Accordingly, the ischemia-reperfusion injury of the PBGs during liver transplantation procedures is associated with the loss of epithelial cells and the development of non-anastomotic biliary strictures [20]. Furthermore, PBGs are activated in primary sclerosing cholangitis (Figure 2(b)) and their hyperplasia has a key role in progression of biliary strictures [21]. Interestingly, proliferating BTSCs can produce Fas-Ligand and modulate inflammatory response by inducing “premature” T-cell apoptosis [22].

BTSC niches extend towards the pancreatic duct system and pancreatic duct glands (PDGs) located along larger pancreatic ducts. The PDGs represent the counterpart of PBGs along the biliary tree [17] and contain a population of committed pancreatic progenitor cells [17, 23]. The anatomical features shared by biliary tree and pancreatic duct system are in touch with the similarities in pathologies affecting these organs, thus suggesting a novel approach to the study of biliary tract's pathophysiology [24].

## 3. HpSCs Participate in the Regeneration of Liver Parenchyma

**Figure 3:**
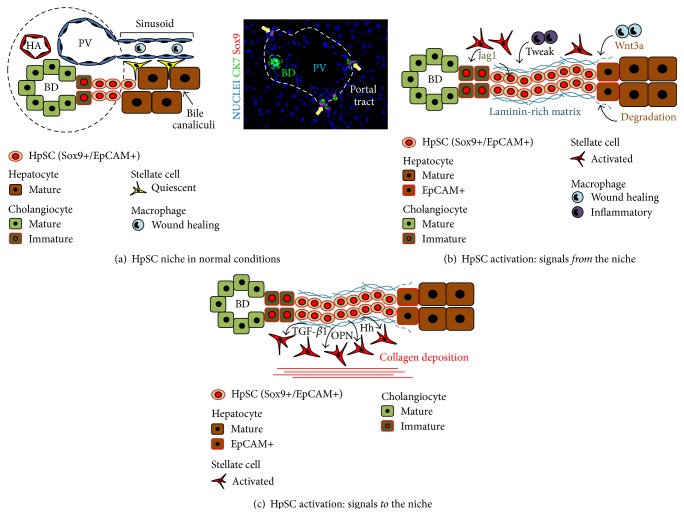
Niche of hepatic stem/progenitor cells (HpSCs). (a) The cartoon shows the HpSC niche in adult liver. The HpSC niche is located within the smaller branches of biliary tree at the interface between portal tract and hepatic parenchyma. The niche is composed of the stem cells in combination with hepatic stellate cell precursors and Kupffer cells (resident macrophages). In the right side image, normal adult human liver: immunofluorescence for Sox9 and cytokeratin (CK) 7 (Original Magnification: 20x); CK7+/Sox9+ HpSCs (arrows) are present in canals of Hering and bile ductules. BD: bile duct; HA: hepatic artery; PV: portal vein. (b-c) Cartoons showing HpSC niche activation in liver diseases. (b) The HpSC response is surrounded by a specialized niche, composed of precursors to hepatic stellate and to endothelial cells and macrophages and of a matrix rich in laminin, hyaluronans, types III and IV collagens, and minimally sulfated proteoglycans. The microenvironment of such a niche maintains the stem/progenitor/biliary phenotype and inhibits hepatocyte differentiation; the transition of the niche matrix environment to one with minimal hyaluronans, less laminin, and an increase in more highly sulfated proteoglycans is a necessary step to start the differentiation into a hepatocyte (or cholangiocyte) phenotype. Cells of mesenchymal origin and macrophages can produce a variety of signals able to drive HpSC responses. Inflammatory macrophages can secrete TNF-like weak inducer of apoptosis (TWEAK) sustaining the expansion of undifferentiated HpSCs; contrarily, tissue-repairing macrophages are able to activate canonical Wnt pathway in HpSCs, triggering their differentiation towards hepatocytes. Activated hepatic stellate cells can secrete Jagged1, thus activating Notch signaling in HpSCs, and also release type I collagen promoting biliary specification. (c) Cartoon showing the profibrogenic loop induced by HpSC activation. HpSCs could activate the liver MF pool via Hedgehog (Hh) pathway, Osteopontin (OPN), and transforming growth factor-*β*1 (TGF-*β*1), thus inducing collagen-I deposition.

Hepatoblasts (HBs) and their precursors, hepatic stem cells (HpSCs), in humans and rodents, and rodent HpSC descendants, called oval cells (Figure 3(a)), are capable of differentiating to mature hepatocytes and cholangiocytes [25]. During embryological development, a ring of cells called the ductal plate forms around the periportal mesenchyme at the portal triads and consists of HpSCs (SOX9+, SOX17+ CK19+, EpCAM+, NCAM+, and AFP−) and HBs (SOX9+, CK19+, EpCAM+, ICAM-1+, and AFP+) [26]. Ductal plate cells have stem cell properties (self-renewal and differentiative capabilities) [27]. The ductal plates transition to become the canals of Hering in pediatric and adult liver and represent the HpSCs niche [28]. Ductal plate cells give also rise to cholangiocytes of interlobular BDs and to diploid hepatocytes [28]. Interestingly, bile duct formations started with asymmetrical ductal structures, partly lined by cholangiocytes and by HBs [29]; then, the HBs lining asymmetrical ducts differentiated to cholangiocytes, with the formation of symmetrical ducts lined only by cholangiocytes [29] and small numbers of HBs tethered to or adjacent to the canals of Hering [27].

In pediatric and adult livers, the normal turnover is accomplished by a combination of diploid adult hepatocytes and cholangiocytes. Interestingly, Axin2+ hepatocytic progenitors found pericentrally participate in the physiological turnover of hepatocytes in mice in quiescence or following minor injuries resulting in mild regenerative demands [12]. On the other side, a preexisting population of Sox9+ periportal hepatocytes (so-called hybrid hepatocytes) can undergo extensive proliferation and replenish liver mass after chronic injuries [30]. Therefore, distinct subpopulation of adult hepatocytes contribute to hepatic regeneration in both homeostasis and injury [31]. In keeping, any participation/activation of HpSCs or BTSCs is sufficiently minor as to be invisible in analyses of homeostatic maintenance of liver parenchyma [32].

Using an* inducible* Cre technology under the control of the Sox9 transcriptional control elements, Furuyama et al. found that Sox9-positive stem/progenitor cells indeed participate in mouse liver homeostatic regeneration [33]. By contrast, other authors using Cre technologies with other genes and in all murine models found that experimental injuries resulted in regenerative demands that cause the stem/progenitors to give rise to only a few percent of the adult parenchymal cells, ones always in the periportal area [34–36]. In particular Español-Suñer et al. [34] and Rodrigo-Torres et al. [36] traced HpSCs in several experimental models of liver injuries in mice using Osteopontin (OPN) or hepatocyte nuclear factor (HNF)1*β*, respectively. Both manuscripts demonstrated that HpSCs contribute minimally to parenchymal turnover during choline-deficient, ethionine-supplemented diet (CDE) while they do not contribute at all in other models of liver injury such as 2/3 partial hepatectomy, bile duct ligation (BDL), carbon tetrachloride intoxication, and 3,5-diethoxycarbonyl-1,4-dihydrocollidine diet (DDC). Moreover, the clonal analysis of Sox9+ cells demonstrated that Sox9+ ductal progenitor cells give rise to clonal oval cells but rarely produce hepatocytes in murine models of liver injuries [37]. In keeping, parallel studies using a hepatocyte fate-tracing model based on transthyretin (TTR) gene demonstrated that mature (TTR+) hepatocytes are the main cells responsible for replacing damaged hepatocytes in experimental injuries [35, 38]. The results were confirmed by genetic and nucleoside analog-based studies to mark and track the origin and contribution of various cell populations to liver regeneration [39]. Interestingly, mature hepatocytes can undergo reversible ductal metaplasia in response to injury and contribute to restoration of the hepatocyte mass [40, 41].

We hypothesize that the remarkably high levels of polyploidy in 3-4-week-old mice, levels of 95–97% or more, with ploidy profiles from 4N to 32N in the liver plates, may restrict the contributions by the BTSCs and HpSCs to the small numbers of diploid cells, all of them located adjacent to the biliary tree. In addition, there is a new report showing that there are committed progenitor niches located pericentrally and containing diploid, Axin2+ hepatocytic progenitors that contribute to normal liver turnover by replacing senescing, mature, polyploid hepatocytes [12]. The relationship of these cells to those of BTSCs and HpSCs is yet to be defined. This new study overcomes the past ones that suggested that newly formed hepatocytes derived only from preexisting hepatocytes [38–41]. Instead, it is now plausible that the interpretations will be altered to give recognition to the Axin2+ unipotent hepatocytic progenitors. A similar reinterpretation is likely for the prior report that hepatocytes can undergo reversible ductal metaplasia, expand as ducts, and contribute to the restoration of the hepatocyte mass in response to injury [42]. Increasingly, the findings are coalescing and reinforcing the prevailing concept that stem/progenitors are the major sources of turnover in mild to extensive forms of liver regeneration.

These past controversies were due in part to the potential pitfalls in lineage tracing that include the choice of which gene is used for lineage tracing, now shown to be critical in defining the results [43], in the dominance in murine livers of highly polyploid hepatocytes [44], and in experimental injury models (such as partial hepatectomy and choline-deficient, ethionine supplemented diet), which do not determine a complete blockade of hepatocyte replication [45, 46]. In this latter regard, an elegant model in zebrafish demonstrated that the extent of hepatocyte injury is fundamental to recruit biliary cells participating in parenchymal recovery after injuries; severe hepatocyte ablation is necessary to elicit the extensive contribution of cells of biliary origin to hepatocyte mass restoration [42]. Recently, Lu and associates developed a mouse model in which the E3 ubiquitin ligase Mdm2 deletion in hepatocytes causes apoptosis, necrosis, and senescence in nearly all hepatocytes [43]. In this model, a florid HpSC activation appeared and was necessary for survival and complete functional liver reconstitution [43].

Complementing these findings are those by Kaneko et al. in which the biliary tree was shown to possess unique architectural and structural flexibilities and responses contributing to maintaining liver homeostasis and in reactions to injuries [44]. Thus, liver injuries determine dynamic structural remodeling of the biliary tree, which corresponds to the pattern of parenchymal cell damage [44]. Chronic damage of pericentral hepatocytes triggers the expansion of biliary branches from the periportal zone towards the injured pericentral area [44]. Future studies are required to learn if biliary tree responses give rise to the Axin2+ hepatocytic progenitors found pericentrally.

Functionally, HpSCs have been further investigated by several* in vitro* assays, which have provided strong evidences of stemness and differentiative potentiality [45]. In this regard, HpSCs were isolated from fetal and adult human livers on the basis of Epithelial Cell Adhesion Molecule (EpCAM) expression [27, 46]. Moreover, HpSCs can be distinctly separated from hepatoblasts by sorting for cells coexpressing EpCAM and neural cell adhesion molecule (NCAM), whereas, HBs are selectively isolated by coexpression for EpCAM and intercellular adhesion molecule (ICAM) [26, 27]. More recently, a single Lgr5+ hepatic stem cell expanded to form epithelial spheroids* in vitro* and was able to differentiate into functional hepatocytes* in vitro* and* in vivo* [47]. Organoids, floating aggregates of epithelia and mesenchymal cells, containing Lgr5+ cells were able to be expanded* ex vivo* and to give rise to hepatocytes and cholangiocytes [48]. In parallel, long-term expansion* ex vivo* of adult bile duct-derived stem/progenitors yielded cells able to lineage restrict into hepatocytes, cholangiocytes, and pancreatic islets [13, 14, 17]. Their potential for giving rise to acinar cells is yet to be examined.

## 4. Role of HpSCs in Human Liver Regeneration

If Axin2+ hepatocytic progenitor niches exist in humans, whatever distinctions they have functionally with HpSC niches in human livers remain to be clarified, and the HpSCs have already been shown actively to contribute to liver regeneration in human diseases [49]. This is due, in part, to the fact that human liver diseases are characterized by a severe and progressive impairment of hepatocyte or cholangiocyte proliferation [50]. Proliferative capabilities of mature liver parenchymal cells are limited and become exhausted due to chronic damage and prolonged cell death [50]. This is in accordance with the increase of proliferative cellular senescence commonly described in hepatocytes [51] in end-stage chronic liver pathologies. Moreover, specific insults lead to the arrest of the hepatocyte cell cycle, such as iron loading in hemochromatosis [52] or oxidative stress in non-alcoholic fatty liver disease (NAFLD) [51, 53]. In parallel, apoptosis of cholangiocytes [54], cellular senescence [55], and a senescence-associated secretory phenotype [56] lead to the production of proinflammatory cytokines and chemokines that may modify the milieu of the bile duct and then trigger fibroinflammatory responses in human cholangiopathies. Consequently, the HpSC compartment is activated and cells proliferate in all human liver diseases [50].

**Figure 4:**
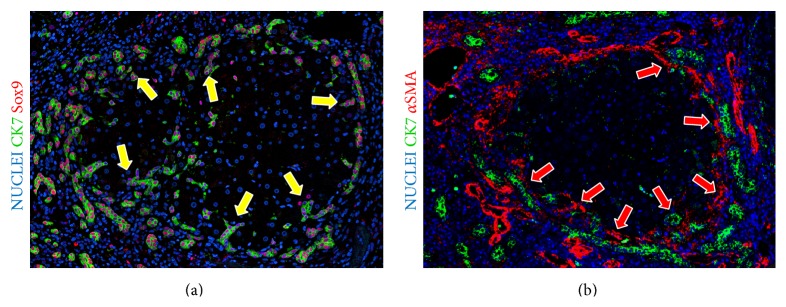
Activation of hepatic stem/progenitor cell (HpSCs) niche in liver diseases. (a) Primary biliary cirrhosis. Immunofluorescence for cytokeratin (CK) 7 and *α*-smooth muscle actin (*α*-SMA); Original Magnification: 10x. The activation of HpSCs is characterized by the appearance of ductular reactions (DR); DR consists of string of cells with irregular lumina (*reactive ductules*) composed of CK7+/Sox9+ HpSCs (yellow arrow). (b) Primary biliary cirrhosis; immunofluorescence for CK7 and *α*-SMA; Original Magnification: 10x. CK7+ DR is surrounded by activated (fibrogenetic) hepatic stellate cells (red arrows).

Under pathological conditions (Figures 3 and 4), unique epithelial cell populations emerge and give rise to the so-called ductular reaction (DR) [57]. DR represents a trans-amplifying population consisting of strings of cells with irregular lumina (*reactive ductules*) and a highly variable phenotypical profile [58, 59]. Virtually all chronic liver diseases (viral hepatitis, alcoholic/non-alcoholic steatohepatitis, hemochromatosis, and primary biliary cirrhosis) and acute (or acute-on-chronic) liver failure are characterized by the emergence of DRs [50].

Variable phenotypes depend on the etiology and are correlated with the progression of the disease [50, 59]. In chronic biliary diseases such as primary biliary cirrhosis (PBC) and primary sclerosing cholangitis (PSC), DRs are prominent and composed mostly of cells expressing biliary (Cytokeratins 7 and 19), neuroendocrine (NCAM, Chromogranin A), and stem cell markers (Sox9, CD133) [50, 59]. On the other hand, in liver diseases of nonbiliary origin, the cells within reactive ductules show hepatocyte-like features (intermediate hepatocytes) [50, 59]. Interestingly, in viral and alcoholic cirrhosis, newly generated hepatocytes derive from DR and represent the progeny of the HpSCs [60]. These newly generated hepatocytes progressively lose biliary markers but maintain EpCAM (stem/progenitor cell marker) expression [60]. Moreover, the stem/progenitor pathway participates in the formation of human cirrhotic nodules with the morphological sequence of bud maturation [61]. Progeny of the bud sequence may represent up to 70% of hepatocytes in cirrhotic livers [61]. However, bud number is typically reduced in biliary disease in association with duct loss and cholestatic destruction of nascent buds [61].

The most prominent DRs can be encountered in acute massive hepatocellular necrosis (fulminant hepatitis), where DR cells are highly proliferating [59, 62]. In general, signs of differentiation toward hepatocytes are minimal in acute liver failure patients [63] and, when present, are a negative prognostic factor [62]; this is due to the fact that, in acute liver failure, activation of HpSCs is secondary to mature hepatocyte proliferation [63]. Moreover, in acute hepatitis, HpSCs predominantly proliferate rather than differentiate [59], and their differentiation starts not earlier than 1 week after the initial liver injury [63]. Interestingly, in acute-on-chronic liver failure, HpSC activation and differentiation are more prominent in comparison with acute liver failure and in decompensated cirrhosis [63].

Finally, several lines of evidence have indicated that HpSC activation takes part in the regenerative response in alcoholic liver disease [64, 65] and NAFLD [25]. Both in adult [51] and pediatric [53, 66] patients with NAFLD, DR is prominent in steatohepatitis but not in simple steatosis. DR appearance and signs of differentiation are associated with hepatocyte cell cycle arrest and apoptosis, and DR extension is correlated with portal fibrosis [51, 53], inflammation [67], and clinical parameters [68]. In alcoholic hepatitis (AH), HpSC activation is correlated with a favorable clinical outcome [65]. In AH patients, the extent of HpSC expansion is associated with liver disease severity [65]. Interestingly, alcohol abstinence induces a clinical improvement that positively correlates with the expansion of the HpSC pool [65]. Unexpectedly, in the natural course of AH, HpSCs do not differentiate into mature hepatocytes due to signals coming from their niche [64]. Clarification of this is given below.

## 5. HpSC Activation Is Driven by a Specialized Niche

In chronic liver injuries (Figures 3 and 4), the stem/progenitor cell response is surrounded by a specialized niche [69]. This niche furnishes several key signals driving HpSC activity (Figure 3(b)). In the hepatic stem cell niche [70], HpSCs are found in association with angioblasts [27], with precursors to hepatic stellate cells and endothelial cells [71, 72], and with macrophages [69, 70]. The precursors have phenotypic traits overlapping with those of mature stellate cells and endothelia but also are distinct. For examples, the stellate cell precursors minimally express retinoids, whereas these are found in abundance in mature stellate cells; the endothelial cell precursors do not express CD31 (PECAM) that is a distinctive feature of mature endothelia. These precursors release paracrine signals that are important for the maintenance of the stem/progenitors in a quiescent state [72, 73]. These paracrine signals include matrix factors (hyaluronans, types III and IV collagens) [71, 72], minimally sulfated proteoglycans [74], and laminins [75] and soluble signals such as leukemia inhibitory factor (LIF), hepatocyte growth factor (HGF), stromal derived growth factor (SDGF), and epidermal growth factor (EGF) [71, 72]. Use of hyaluronan substrata and a serum-free medium devoid of these soluble signaling molecules enables self-replicative, clonogenic expansion of human and rodent HpSCs [76]. Therefore, the addition of any of these factors to the conditions elicits differentiation of the HpSCs to HBs and then their dramatic expansion [71, 72, 76].

In diseased tissue, there are activated hepatic stellate cells and myofibroblasts (MFs) that produce distinct paracrine signals (e.g., type I collagen, sulfated proteoglycans, and high levels of the cytokines and growth factors) from those of the quiescent stellate cells (e.g., network collagens, minimally sulfated proteoglycans, and LIF) [71, 77].

The HpSCs, MFs, and macrophages produce a variety of signals able to drive the HpSC response [78]. Interestingly, macrophages can produce a variety of cytokines, which have a key role in the prominent expansion of undifferentiated HpSCs [79, 80]. Interestingly, a single injection of unfractionated bone marrow cells in healthy mice is able to induced HpSC proliferation and this is dependent by the macrophage production of TNF-like weak inducer of apoptosis (TWEAK) [79]. Interestingly, TWEAK stimulates HpSC proliferation through its receptor Fn14 and HpSC expansion was significantly reduced in Fn14-null mice or using a blocking anti-TWEAK antibody [80]. In chronic liver diseases, macrophages are able to activate the canonical Wnt pathway in HpSCs triggering their differentiation towards hepatocyte [78]. In biliary diseases, activated stellate cells and MFs can secrete Jagged1 that activates Notch signaling in nearby HpSCs and, along with production of type I collagen, promote their biliary specification [59, 81]. Therefore, Notch and Wnt signaling pathways have a key role in HpSC proliferation and specification [69].

A crucial element for the responses of the HpSCs and HBs is represented by extracellular matrix (ECM) composition [82]. Macrophages, MFs, HpSCs, and HBs have pivotal roles in remodelling ECM through the production of a variety of matrix metalloproteinases and their tissue inhibitors [69] and in the synthesis of specific types of matrix components [73]. The degradation of the collagen matrix by the metalloproteinases, coupled with the production of a laminin-rich niche, leads to HpSC expansion; laminin maintains the stem/progenitor/biliary phenotype and inhibits hepatocyte differentiation [83]. By contrast, the loss of the laminin-rich niche is a necessary step to start the differentiation into a hepatocyte phenotype [82, 83]. Interestingly, in alcoholic hepatitis, livers predominantly express laminin and, consequently, HpSC expansion is inefficient at yielding mature hepatocytes [64].

The interaction of HpSCs with a laminin-rich matrix is promoted by *β*-galactoside-binding lectin galectin-3 (Gal-3) [84]. Interestingly, Gal-3 is able to promote HpSC expansion in an undifferentiated form [84]. Similarly, cell-cell and cell-matrix interaction of unactivated HpSCs are mediated by NCAM, a surface marker found on HpSCs and on both angioblasts and endothelial cell precursors [27, 70, 73, 85]. Subsequent to liver injury, NCAM-positive cells expand and typical NCAM posttranslational modification (polySia) is produced [85]; PolySia weakens cell-cell and cell-matrix interactions, facilitating HpSC migration away from the laminin niche and their subsequent differentiation [85].

In addition to signals passing from the niche to the stem/progenitor cells, there are also signals from the stem/progenitor cells to the niche (Figure 3(c)) [69]. HpSCs can activate stellate/endothelial cells via the Hedgehog (Hh) pathway resulting in release of types of matrix components (e.g., type IV collagen, laminin, syndecans, and glypicans) associated with normal liver regeneration [70, 73, 86]; other key paracrine signals include OPN and transforming growth factor-*β*1 (TGF-*β*1) which induce collagen-I deposition and other matrix components associated with fibrosis by stellate cells and MFs [87, 88]. The OPN synthesized by HpSCs could also have an autocrine role in HpSC expansion and migration (via disruption of cell adhesion) [89]. In chronic pathological conditions, this cellular cross-talk of paracrine signals could be responsible in establishing a profibrogenic loop [57]; in fibrogenesis, MF activation is secondary to the expansion of the HpSC compartment mediated in part by the Hh pathway [90] along with signaling pathways induced by chronic injury [77].

In general, the emerging concept is that the expansion of the HpSC niche (Figure 4(a)) represents an attempt to participate in the regeneration of damaged liver. Unfortunately, the persistent injury and the chronic inflammatory milieu activate profibrogenetic pathways (Figure 4(b)) and lead to deposition of type I collagen and associated matrix components typical of scar formation and of fibrosis [57]. In addition, in several human diseases and experimental settings, the expansion of the HpSC niche is strongly correlated with fibrosis progression [90]; the attenuation of the liver stem/progenitor cell response in experimental settings by the ablation of OPN expression [88] or by using anti-TWEAK antibody [91] is able to prevent fibrogenic response and improve liver regeneration. Interestingly, HpSCs are hypothesized to contribute directly or indirectly to epithelial-to-mesenchymal transitions (EMT), thus influencing the MF pool [92]. The Hh pathway activation has a key role in EMT of HpSCs and ductular cells in cirrhosis [93]. Interestingly, EMT associated with HpSCs can also be driven by several other factors such as the noncanonical Wnt pathway [94] and TGF-*β*1 [95].

## 6. Pathological Aspects and Clinical Perspectives

From a point of view of pathology, distinct subpopulations of mature hepatocytes and stem/progenitor cell compartments are differently activated in the course of normal, quiescent liver biology versus different human pathologies. The Axin2+ hepatocytic progenitors are activated for normal liver turnover [12], and their role in human pathology should be further investigated; the HpSC niche is activated in diseases involving hepatocyte damage and ones involving interlobular BDs following severe liver injury when mature cell senescence develops [31, 69]; by contrast, the BTSC niche is involved in diseases affecting larger intrahepatic and extrahepatic BDs [21]. These aspects have been clearly elucidated comparing stem/progenitor cells responses in different biliary pathologies such as PBC and PSC [21]. PSC affects large intrahepatic and extrahepatic BDs and, in addition, activation of PBGs is triggered [21]. On the other hand, PBC primarily affects interlobular bile ducts and, therefore, PBG hyperplasia is almost absent [21]. In summary, distinct stem/progenitor cell niches are implicated in different forms of pathogenesis. This enables one to analyze the natural course and complications of different liver pathologies leading to a concept of a lineage-specificity of liver diseases.

Investigations of stem/progenitor niches within the biliary tree are acquiring particular relevance in relation to primitive liver cancers (hepatocarcinoma: HCC), fibrolamellar HCC, and cholangiocarcinoma (CCA). Indeed, recent evidences indicate that a subgroup of HCC tumors, such as cytokeratin-19 positive HCC and combined HCC-CCA, may originate from HpSCs [96]. The HpSCs could represent the cell of origin of a subtype of CCA such as cholangiolocarcinoma (CLC) and the so-called mixed-type CCA [97, 98]. On the other hand, PBGs and BTSCs could represent the cell of origin of pure mucin-producing CCAs [99, 100] and fibrolamellar-HCC [9]. In addition, clinical-pathological correlates and risk factors for HCC and CCA support the role of HpSC and BTSC in different subtypes of liver cancers [101].

The presence of stem/progenitor niches within the ducts (intramural glands) and along the surface of ducts (extramural glands) of the biliary tree has important implications in the regenerative medicine for liver and biliary disorders [102]. Actually, cell therapies for liver disease are demanding given the organ shortage for orthotopic liver transplantation [102, 103]. Mature hepatocyte transplantation is limited by several issues, most importantly that the transplantation is associated with complications such as emboli and that the effects are transient [102, 103]. Therefore, other cell types have been proposed and tested in preclinical models or clinical settings [102, 103]. In this context, HpSCs have been indicated as candidate cells for clinical use [103]; HpSCs are long-term expandable and highly stable at the chromosome and structural level [48]; however, to date, few clinical trials have been started or completed [102–104]. In a trial of 25 subjects and 25 controls with decompensated liver cirrhosis due to various causes, subjects received fetal liver-derived EpCAM+ cell infusions into the liver via the hepatic artery and showed improvement in multiple diagnostic and biochemical parameters [105]. Beside decompensated liver cirrhosis, the use of HpSCs in pediatric patients affected by inherited liver inborn errors of metabolism has been tested [106, 107].

Alternatively, the transplantation of cells of mesenchymal origin (such as macrophages, endothelial cells, or mesenchymal stem cells) has been proposed as cell therapy approach to liver cirrhosis to stimulate endogenous regeneration or decrease fibrosis. The peripheral administration of autologous mesenchymal (or hematopoietic) stem cells has been tested in several clinical trials in cirrhosis [102, 108, 109]; these clinical studies of various autologous cells for liver disease have been recently systematically reviewed and to date no convincing benefit has been noted in adequately powered randomized controlled studies [110].

Interestingly, human extrahepatic biliary tree represents a suitable and large source for cell therapy [111]. Recently, preliminary data regarding the infusion of BTSCs in patients with advanced cirrhosis have been reported, representing the basis for forthcoming clinical trials [111].

In general, further studies focusing on the optimal routes of cell transplantation, the need for immunosuppression, and methods to improve the engraftment and proliferation rate of transplanted cells are required [102, 103]. A key point regarding the clinical use of cells with stem cell properties resides in the potential risk of unwanted and unregulated cell growth; taken in consideration that stem/progenitor cells can function as cell of origin for liver tumors, oncogenic risks should be carefully considered when cells are candidate for therapy in humans. To this regard, embryonic stem cells, induced pluripotent stem cells, and induced hepatocyte cells may be phenotypically and genetically unstable* in vivo* over a prolonged period and when transplanted into the damaged liver [112]. Interestingly, extensive analysis of the genetic stability of primary human HpSCs demonstrated that the expanded cells preserve their genetic integrity over months in culture [48]. Moreover, the long-term studies of BTSC in appropriate animal models showed low oncogenetic risk [16]. In general, oncogenic risks should be minimized by screening candidate cells in long-term preclinical studies in animal models in order to evaluate the safety and the absence of oncogenicity of the cells.

## References

[1] Cardinale V., Wang Y., Carpino G. (2012). The biliary tree—a reservoir of multipotent stem cells. *Nature Reviews Gastroenterology and Hepatology*.

[2] Nakanuma Y., Hoso M., Sanzen T., Sasaki M. (1997). Microstructure and development of the normal and pathologic biliary tract in humans, including blood supply. *Microscopy Research and Technique*.

[3] Roskams T. A., Theise N. D., Balabaud C. (2004). Nomenclature of the finer branches of the biliary tree: canals, ductules, and ductular reactions in human livers. *Hepatology*.

[4] Glaser S. S., Gaudio E., Rao A. (2009). Morphological and functional heterogeneity of the mouse intrahepatic biliary epithelium. *Laboratory Investigation*.

[5] Glaser S., Lam I. P., Franchitto A. (2010). Knockout of secretin receptor reduces large cholangiocyte hyperplasia in mice with extrahepatic cholestasis induced by bile duct ligation. *Hepatology*.

[6] Mancinelli R., Franchitto A., Glaser S. (2013). GABA induces the differentiation of small into large cholangiocytes by activation of Ca^2+^/CaMK I-dependent adenylyl cyclase 8. *Hepatology*.

[7] Renzi A., Glaser S., DeMorrow S. (2011). Melatonin inhibits cholangiocyte hyperplasia in cholestatic rats by interaction with MT1 but not MT2 melatonin receptors. *American Journal of Physiology—Gastrointestinal and Liver Physiology*.

[8] Roskams T., Desmet V. (2008). Embryology of extra- and intrahepatic bile ducts, the ductal plate. *Anatomical Record*.

[9] Oikawa T., Wauthier E., Dinh T. A. (2015). Model of fibrolamellar hepatocellular carcinomas reveals striking enrichment in cancer stem cells. *Nature Communications*.

[10] Itoh T., Miyajima A. (2014). Liver regeneration by stem/progenitor cells. *Hepatology*.

[11] Turner R., Lozoya O., Wang Y. (2011). Human hepatic stem cell and maturational liver lineage biology. *Hepatology*.

[12] Wang B., Zhao L., Fish M., Logan C. Y., Nusse R. (2015). Self-renewing diploid Axin2^+^ cells fuel homeostatic renewal of the liver. *Nature*.

[13] Carpino G., Cardinale V., Onori P. (2012). Biliary tree stem/progenitor cells in glands of extrahepatic and intraheptic bile ducts: an anatomical in situ study yielding evidence of maturational lineages. *Journal of Anatomy*.

[14] Cardinale V., Wang Y., Carpino G. (2011). Multipotent stem/progenitor cells in human biliary tree give rise to hepatocytes, cholangiocytes, and pancreatic islets. *Hepatology*.

[15] Terada T., Morita T., Hoso M., Nakanuma Y. (1994). Pancreatic enzymes in the epithelium of intrahepatic large bile ducts and in hepatic bile in patients with extrahepatic bile duct obstruction. *Journal of Clinical Pathology*.

[16] Carpino G., Cardinale V., Gentile R. (2014). Evidence for multipotent endodermal stem/progenitor cell populations in human gallbladder. *Journal of Hepatology*.

[17] Wang Y., Lanzoni G., Carpino G. (2013). Biliary tree stem cells, precursors to pancreatic committed progenitors: evidence for possible life-long pancreatic organogenesis. *STEM CELLS*.

[18] Dipaola F., Shivakumar P., Pfister J., Walters S., Sabla G., Bezerra J. A. (2013). Identification of intramural epithelial networks linked to peribiliary glands that express progenitor cell markers and proliferate after injury in mice. *Hepatology*.

[19] Sutton M. E., Op den Dries S., Koster M. H., Lisman T., Gouw A. S. H., Porte R. J. (2012). Regeneration of human extrahepatic biliary epithelium: the peribiliary glands as progenitor cell compartment. *Liver International*.

[20] Op den Dries S., Westerkamp A. C., Karimian N. (2014). Injury to peribiliary glands and vascular plexus before liver transplantation predicts formation of non-anastomotic biliary strictures. *Journal of Hepatology*.

[21] Carpino G., Cardinale V., Renzi A. (2015). Activation of biliary tree stem cells within peribiliary glands in primary sclerosing cholangitis. *Journal of Hepatology*.

[22] Riccio M., Carnevale G., Cardinale V. (2014). The Fas/Fas ligand apoptosis pathway underlies immunomodulatory properties of human biliary tree stem/progenitor cells. *Journal of Hepatology*.

[23] Yamaguchi J., Liss A. S., Sontheimer A. (2015). Pancreatic duct glands (PDGs) are a progenitor compartment responsible for pancreatic ductal epithelial repair. *Stem Cell Research*.

[24] Nakanuma Y. (2010). A novel approach to biliary tract pathology based on similarities to pancreatic counterparts: is the biliary tract an incomplete pancreas?. *Pathology International*.

[25] Carpino G., Renzi A., Onori P., Gaudio E. (2013). Role of hepatic progenitor cells in nonalcoholic fatty liver disease development: cellular cross-talks and molecular networks. *International Journal of Molecular Sciences*.

[26] Schmelzer E., Wauthier E., Reid L. M. (2006). The phenotypes of pluripotent human hepatic progenitors. *STEM CELLS*.

[27] Schmelzer E., Zhang L., Bruce A. (2007). Human hepatic stem cells from fetal and postnatal donors. *The Journal of Experimental Medicine*.

[28] Carpentier R., Suer R. E., Van Hul N. (2011). Embryonic ductal plate cells give rise to cholangiocytes, periportal hepatocytes, and adult liver progenitor cells. *Gastroenterology*.

[29] Antoniou A., Raynaud P., Cordi S. (2009). Intrahepatic bile ducts develop according to a new mode of tubulogenesis regulated by the transcription factor SOX9. *Gastroenterology*.

[30] Font-Burgada J., Shalapour S., Ramaswamy S. (2015). Hybrid periportal hepatocytes regenerate the injured liver without giving rise to cancer. *Cell*.

[31] Bird T., Forbes S. (2015). Two fresh streams to fill the liver’s hepatocyte pool. *Cell Stem Cell*.

[32] Alison M. R., Golding M. H. C., Sarraf C. E. (1996). Pluripotential liver stem cells: facultative stem cells located in the biliary tree. *Cell Proliferation*.

[33] Furuyama K., Kawaguchi Y., Akiyama H. (2011). Continuous cell supply from a Sox9-expressing progenitor zone in adult liver, exocrine pancreas and intestine. *Nature Genetics*.

[34] Español-Suñer R., Carpentier R., Van Hul N. (2012). Liver progenitor cells yield functional hepatocytes in response to chronic liver injury in mice. *Gastroenterology*.

[35] Malato Y., Naqvi S., Schürmann N. (2011). Fate tracing of mature hepatocytes in mouse liver homeostasis and regeneration. *The Journal of Clinical Investigation*.

[36] Rodrigo-Torres D., Affò S., Coll M. (2014). The biliary epithelium gives rise to liver progenitor cells. *Hepatology*.

[37] Tarlow B. D., Finegold M. J., Grompe M. (2014). Clonal tracing of Sox9^+^ liver progenitors in mouse oval cell injury. *Hepatology*.

[38] Schaub J. R., Malato Y., Gormond C., Willenbring H. (2014). Evidence against a stem cell origin of new hepatocytes in a common mouse model of chronic liver injury. *Cell Reports*.

[39] Yanger K., Knigin D., Zong Y. (2014). Adult hepatocytes are generated by self-duplication rather than stem cell differentiation. *Cell Stem Cell*.

[40] Tarlow B. D., Pelz C., Naugler W. E. (2014). Bipotential adult liver progenitors are derived from chronically injured mature hepatocytes. *Cell Stem Cell*.

[41] Yimlamai D., Christodoulou C., Galli G. G. (2014). Hippo pathway activity influences liver cell fate. *Cell*.

[42] Choi T.-Y., Ninov N., Stainier D. Y. R., Shin D. (2014). Extensive conversion of hepatic biliary epithelial cells to hepatocytes after near total loss of hepatocytes in zebrafish. *Gastroenterology*.

[43] Lu W. Y., Bird T. G., Boulter L. (2015). Hepatic progenitor cells of biliary origin with liver repopulation capacity. *Nature Cell Biology*.

[44] Kaneko K., Kamimoto K., Miyajima A., Itoh T. (2015). Adaptive remodeling of the biliary architecture underlies liver homeostasis. *Hepatology*.

[45] Miyajima A., Tanaka M., Itoh T. (2014). Stem/progenitor cells in liver development, homeostasis, regeneration, and reprogramming. *Cell Stem Cell*.

[46] Turner R. A., Wauthier E., Lozoya O. (2013). Successful transplantation of human hepatic stem cells with restricted localization to liver using hyaluronan grafts. *Hepatology*.

[47] Huch M., Dorrell C., Boj S. F. (2013). *In vitro* expansion of single Lgr5^+^ liver stem cells induced by Wnt-driven regeneration. *Nature*.

[48] Huch M., Gehart H., van Boxtel R. (2015). Long-term culture of genome-stable bipotent stem cells from adult human liver. *Cell*.

[49] Theise N. D., Kuwahara R. (2007). The tissue biology of ductular reactions in human chronic liver disease. *Gastroenterology*.

[50] Gouw A. S. H., Clouston A. D., Theise N. D. (2011). Ductular reactions in human liver: diversity at the interface. *Hepatology*.

[51] Richardson M. M., Jonsson J. R., Powell E. E. (2007). Progressive fibrosis in nonalcoholic steatohepatitis: association with altered regeneration and a ductular reaction. *Gastroenterology*.

[52] Wood M. J., Gadd V. L., Powell L. W., Ramm G. A., Clouston A. D. (2014). Ductular reaction in hereditary hemochromatosis: the link between hepatocyte senescence and fibrosis progression. *Hepatology*.

[53] Nobili V., Carpino G., Alisi A. (2012). Hepatic progenitor cells activation, fibrosis, and adipokines production in pediatric nonalcoholic fatty liver disease. *Hepatology*.

[54] Onori P., Alvaro D., Floreani A. (2007). Activation of the IGF1 system characterizes cholangiocyte survival during progression of primary biliary cirrhosis. *The Journal of Histochemistry and Cytochemistry*.

[55] Nakanuma Y., Sasaki M., Harada K. (2015). Autophagy and senescence in fibrosing cholangiopathies. *Journal of Hepatology*.

[56] Tabibian J. H., O'Hara S. P., Splinter P. L., Trussoni C. E., Larusso N. F. (2014). Cholangiocyte senescence by way of N-Ras activation is a characteristic of primary sclerosing cholangitis. *Hepatology*.

[57] Williams M. J., Clouston A. D., Forbes S. J. (2014). Links between hepatic fibrosis, ductular reaction, and progenitor cell expansion. *Gastroenterology*.

[58] Gaudio E., Carpino G., Cardinale V., Franchitto A., Onori P., Alvaro D. (2009). New insights into liver stem cells. *Digestive and Liver Disease*.

[59] Spee B., Carpino G., Schotanus B. A. (2010). Characterisation of the liver progenitor cell niche in liver diseases: potential involvement of Wnt and Notch signalling. *Gut*.

[60] Yoon S.-M., Gerasimidou D., Kuwahara R. (2011). Epithelial cell adhesion molecule (EpCAM) marks hepatocytes newly derived from stem/progenitor cells in humans. *Hepatology*.

[61] Stueck A. E., Wanless I. R. (2015). Hepatocyte buds derived from progenitor cells repopulate regions of parenchymal extinction in human cirrhosis. *Hepatology*.

[62] Katoonizadeh A., Nevens F., Verslype C., Pirenne J., Roskams T. (2006). Liver regeneration in acute severe liver impairment: a clinicopathological correlation study. *Liver International*.

[63] Rastogi A., Maiwall R., Bihari C., Trehanpati N., Pamecha V., Sarin S. K. (2014). Two-tier regenerative response in liver failure in humans. *Virchows Archiv*.

[64] Dubuquoy L., Louvet A., Lassailly G. (2015). Progenitor cell expansion and impaired hepatocyte regeneration in explanted livers from alcoholic hepatitis. *Gut*.

[65] Lanthier N., Rubbia-Brandt L., Lin-Marq N. (2015). Hepatic cell proliferation plays a pivotal role in the prognosis of alcoholic hepatitis. *Journal of Hepatology*.

[66] Nobili V., Carpino G., Alisi A. (2014). Role of docosahexaenoic acid treatment in improving liver histology in pediatric nonalcoholic fatty liver disease. *PLoS ONE*.

[67] Gadd V. L., Skoien R., Powell E. E. (2014). The portal inflammatory infiltrate and ductular reaction in human nonalcoholic fatty liver disease. *Hepatology*.

[68] Nobili V., Alisi A., Cutrera R. (2015). Altered gut-liver axis and hepatic adiponectin expression in OSAS: novel mediators of liver injury in paediatric non-alcoholic fatty liver. *Thorax*.

[69] Boulter L., Lu W.-Y., Forbes S. J. (2013). Differentiation of progenitors in the liver: a matter of local choice. *The Journal of Clinical Investigation*.

[70] Zhang L., Theise N., Chua M., Reid L. M. (2008). The stem cell niche of human livers: symmetry between development and regeneration. *Hepatology*.

[71] Wang Y., Yao H.-L., Cui C.-B. (2010). Paracrine signals from mesenchymal cell populations govern the expansion and differentiation of human hepatic stem cells to adult liver fates. *Hepatology*.

[72] Kubota H., Yao H.-L., Reid L. M. (2007). Identification and characterization of vitamin A-storing cells in fetal liver: implications for functional importance of hepatic stellate cells in liver development and hematopoiesis. *STEM CELLS*.

[73] Wang Y., Cui C.-B., Yamauchi M. (2011). Lineage restriction of human hepatic stem cells to mature fates is made efficient by tissue-specific biomatrix scaffolds. *Hepatology*.

[74] Hayes A. J., Tudor D., Nowell M. A., Caterson B., Hughes C. E. (2008). Chondroitin sulfate sulfation motifs as putative biomarkers for isolation of articular cartilage progenitor cells. *The Journal of Histochemistry and Cytochemistry*.

[75] Couvelard A., Bringuier A. F., Dauge M. C. (1998). Expression of integrins during liver organogenesis in humans. *Hepatology*.

[76] Harrill J. A., Parks B. B., Wauthier E., Rowlands J. C., Reid L. M., Thomas R. S. (2015). Lineage-dependent effects of aryl hydrocarbon receptor agonists contribute to liver tumorigenesis. *Hepatology*.

[77] Lee Y. A., Wallace M. C., Friedman S. L. (2015). Pathobiology of liver fibrosis: a translational success story. *Gut*.

[78] Boulter L., Govaere O., Bird T. G. (2012). Macrophage-derived Wnt opposes Notch signaling to specify hepatic progenitor cell fate in chronic liver disease. *Nature Medicine*.

[79] Bird T. G., Lu W.-Y., Boulter L. (2013). Bone marrow injection stimulates hepatic ductular reactions in the absence of injury via macrophage-mediated TWEAK signaling. *Proceedings of the National Academy of Sciences of the United States of America*.

[80] Jakubowski A., Ambrose C., Parr M. (2005). TWEAK induces liver progenitor cell proliferation. *The Journal of Clinical Investigation*.

[81] Kim K. H., Chen C. C., Alpini G., Lau L. F. (2015). CCN1 induces hepatic ductular reaction through integrin alphavbeta(5)-mediated activation of NF-kappaB. *The Journal of Clinical Investigation*.

[82] Lorenzini S., Bird T. G., Boulter L. (2010). Characterisation of a stereotypical cellular and extracellular adult liver progenitor cell niche in rodents and diseased human liver. *Gut*.

[83] Kallis Y. N., Robson A. J., Fallowfield J. A. (2011). Remodelling of extracellular matrix is a requirement for the hepatic progenitor cell response. *Gut*.

[84] Hsieh W.-C., Mackinnon A. C., Lu W.-Y. (2015). Galectin-3 regulates hepatic progenitor cell expansion during liver injury. *Gut*.

[85] Tsuchiya A., Lu W. Y., Weinhold B. (2014). Polysialic acid/neural cell adhesion molecule modulates the formation of ductular reactions in liver injury. *Hepatology*.

[86] Sicklick J. K., Li Y.-X., Melhem A. (2006). Hedgehog signaling maintains resident hepatic progenitors throughout life. *The American Journal of Physiology—Gastrointestinal and Liver Physiology*.

[87] Wang X., Lopategi A., Ge X. (2014). Osteopontin induces ductular reaction contributing to liver fibrosis. *Gut*.

[88] Coombes J. D., Swiderska-Syn M., Dollé L. (2015). Osteopontin neutralisation abrogates the liver progenitor cell response and fibrogenesis in mice. *Gut*.

[89] Liu Y., Cao L., Chen R. (2015). Osteopontin promotes hepatic progenitor cell expansion and tumorigenicity via activation of *β*-catenin in mice. *STEM CELLS*.

[90] Grzelak C. A., Martelotto L. G., Sigglekow N. D. (2014). The intrahepatic signalling niche of hedgehog is defined by primary cilia positive cells during chronic liver injury. *Journal of Hepatology*.

[91] Kuramitsu K., Sverdlov D. Y., Liu S. B. (2013). Failure of fibrotic liver regeneration in mice is linked to a severe fibrogenic response driven by hepatic progenitor cell activation. *The American Journal of Pathology*.

[92] Xie G., Diehl A. M. (2013). Evidence for and against epithelial-to-mesenchymal transition in the liver. *American Journal of Physiology—Gastrointestinal and Liver Physiology*.

[93] Syn W.-K., Jung Y., Omenetti A. (2009). Hedgehog-mediated epithelial-to-mesenchymal transition and fibrogenic repair in nonalcoholic fatty liver disease. *Gastroenterology*.

[94] Chen J., Zhang X., Xu Y. (2015). Hepatic progenitor cells contribute to the progression of 2-acetylaminofluorene/carbon tetrachloride-induced cirrhosis via the non-canonical Wnt pathway. *PLoS One*.

[95] Wang P., Yang A.-T., Cong M. (2015). EGF suppresses the initiation and drives the reversion of TGF-*β*1-induced transition in hepatic oval cells showing the plasticity of progenitor cells. *Journal of Cellular Physiology*.

[96] Govaere O., Komuta M., Berkers J. (2014). Keratin 19: a key role player in the invasion of human hepatocellular carcinomas. *Gut*.

[97] Komuta M., Govaere O., Vandecaveye V. (2012). Histological diversity in cholangiocellular carcinoma reflects the different cholangiocyte phenotypes. *Hepatology*.

[98] Komuta M., Spee B., Vander Borght S. (2008). Clinicopathological study on cholangiolocellular carcinoma suggesting hepatic progenitor cell origin. *Hepatology*.

[99] Cardinale V., Wang Y., Carpino G., Reid L. M., Gaudio E., Alvaro D. (2012). Mucin-producing cholangiocarcinoma might derive from biliary tree stem/progenitor cells located in peribiliary glands. *Hepatology*.

[100] Rizvi S., Gores G. J. (2013). Pathogenesis, diagnosis, and management of cholangiocarcinoma. *Gastroenterology*.

[101] Cardinale V., Semeraro R., Torrice A. (2010). Intra-hepatic and extra-hepatic cholangiocarcinoma: new insight into epidemiology and risk factors. *World Journal of Gastrointestinal Oncology*.

[102] Lanzoni G., Oikawa T., Wang Y. (2013). Concise review: clinical programs of stem cell therapies for liver and pancreas. *STEM CELLS*.

[103] Forbes S. J., Gupta S., Dhawan A. (2015). Cell therapy for liver disease: from liver transplantation to cell factory. *Journal of Hepatology*.

[104] Gridelli B., Vizzini G., Pietrosi G. (2012). Efficient human fetal liver cell isolation protocol based on vascular perfusion for liver cell-based therapy and case report on cell transplantation. *Liver Transplantation*.

[105] Khan A. A., Shaik M. V., Parveen N. (2010). Human fetal liver-derived stem cell transplantation as supportive modality in the management of end-stage decompensated liver cirrhosis. *Cell Transplantation*.

[106] Defresne F., Tondreau T., Stéphenne X. (2014). Biodistribution of adult derived human liver stem cells following intraportal infusion in a 17-year-old patient with glycogenosis type 1A. *Nuclear Medicine and Biology*.

[107] Sokal E. M. (2014). Treating inborn errors of liver metabolism with stem cells: current clinical development. *Journal of Inherited Metabolic Disease*.

[108] Zekri A. R., Salama H., Medhat E. (2015). The impact of repeated autologous infusion of haematopoietic stem cells in patients with liver insufficiency. *Stem Cell Research & Therapy*.

[109] Andreone P., Catani L., Margini C. (2015). Reinfusion of highly purified CD133^+^ bone marrow-derived stem/progenitor cells in patients with end-stage liver disease: a phase I clinical trial. *Digestive and Liver Disease*.

[110] Moore J. K., Stutchfield B. M., Forbes S. J. (2014). Systematic review: the effects of autologous stem cell therapy for patients with liver disease. *Alimentary Pharmacology and Therapeutics*.

[111] Cardinale V., Carpino G., Gentile R. (2014). Transplantation of human fetal biliary tree stem/progenitor cells into two patients with advanced liver cirrhosis. *BMC Gastroenterology*.

[112] Bayart E., Cohen-Haguenauer O. (2013). Technological overview of iPS induction from human adult somatic cells. *Current Gene Therapy*.

